# The Association between Cardiovascular Disease Risk and Parental Educational Level in Portuguese Children

**DOI:** 10.3390/ijerph9124311

**Published:** 2012-11-27

**Authors:** Michael J. Duncan, Susana Vale, Maria Paula Santos, José Carlos Ribeiro, Jorge Mota

**Affiliations:** 1 Sport and Exercise Applied Research Group, Faculty of Health and Life Sciences, Coventry University, Priory Street, Coventry CV11 5FB, UK; 2 Research Centre in Physical Activity Health and Leisure, Faculty of Sports Sciences and Physical Education, University of Porto, Porto 4200-450, Portugal; Email: susanavale@hotmail.com (S.V.); msantos@fade.up.pt (M.P.S.); jribeiro@fade.up.pt (J.C.R.); jmota@fade.up.pt (J.M.)

**Keywords:** obesity, metabolic syndrome, parental educational level

## Abstract

The aim of this study was to examine any differences in cardiovascular disease (CVD) risk in Portuguese children split by parental educational level. A cross-sectional school-based study was conducted in 2011 on 359 Portuguese children (202 girls and 157 boys) aged 10 to 17 years (mean age ± SD = 13.9 ± 1.98 years). Height and body mass were assessed to determine body mass index (BMI). Parental education level (PEL) was used as a surrogate for socioeconomic status (SES). Capillary blood sampling was used to determine: Total Cholesterol (TC), Triglycerides (TG), Fasting Glucos (GLUC), High and Low Density Lipoprotein (HDL/LDL). These measurements were combined with measures of systolic blood pressure and cardiorespiratory fitness as z-scores. CVD risk was constructed by summing the z-scores. Analysis of covariance, controlling for BMI, indicated that CVD risk was significantly different across PEL groups (*p* = 0.01), with CVD risk score being significantly lower in low (*p* = 0.04) and middle (*p* = 0.008) PEL groups, compared to high PEL. Moreover, the covariate, BMI was also significant (*p* = 0.0001, β = 0.023), evidencing a significant positive association between BMI and CVD risk, with higher BMI associated with greater CVD risk. In Portuguese children, significantly greater CVD risk was found for children of high PEL, while higher BMI was associated with greater CVD risk.

## 1. Introduction

Increasing incidence of type 2 diabetes, metabolic abnormality and clustering of cardiovascular disease (CVD) risk factors are worldwide public health concerns [1]. Excess weight gain appears to be a key factor, although the relationships are not straightforward [2]. Prior research has examined the associations between CVD risk and a number of other variables, including active commuting [3], screen time [4], physical fitness [5] and physical activity [3]. The predominant approach taken in these studies has been to assess a range of variables independently associated with cardiovascular disease or metabolic abnormality, including aerobic fitness, resting blood pressure, waist circumference, body fatness, blood lipids, blood glucose and insulin [6]. A number of studies have also attempted to provide a CVD risk score by converting all these physiological measures to z-scores and summing them to provide a single measure with high scores indicating increased CVD risk [3,7]. However, although this approach has its merits, the literature in this area is mixed, partially due to the range of different dependant variables employed to construct the summed CVD risk scores. Friedemann *et al.* [6] recently performed a systematic review of CVD in children and adolescents and identified a range of variables which could be employed as measures of CVD risk, including objectively assessed weight status, resting blood pressure, blood cholesterol (HDL, LDL, Total Cholesterol, Triglyceride), fasting glucose, fasting insulin, Homeostasis Model Assessment of Insulin Resistance, carotid intima media thickness and left ventricular mass [6]. They concluded that body mass index was independently associated with CVD risk in children and that the continuous association between body mass index and risk parameters for CVD is an area for future research, particularly if the change in risk parameters perunit increase of body mass index can be established [6]. However, they also go on to note that the literature in this area is hampered by lack of consensus in cut-off points, leading to misclassification of children’s disease risk and highlight that research examining the association between weight status and CVD risk rather than simply the association between weight status and individual risk factors for CVD is needed.

The need for additional data examining the impact of weight status on CVD risk is important because a number of prior studies have not fully characterized the association of environmental, social and economical factors on CVD risk in children [7]. Sociodemographic variables, including socioeconomic status (SES), have been highlighted as important factors in the development of CVD in both adults and children [8]. The degree to which SES influences CVD risk is unclear, and data on this topic are equivocal, with some studies suggesting an inverse relationship between CVD risk and deprivation, others suggesting the opposite and others identifying no association [9]. In some ways the equivocal nature of the literature in the area is understandable. Literature has suggested that low socioeconomic status is associated with greater CVD risk because low SES is associated with lower income, poorer career prospects and greater risk of unemployment [10]. All of these factors determine an individual’s capacity to consume goods and services, including purchase of high quality food and health care [10]. Likewise, the lower level of education associated with low SES results in poorer knowledge regarding healthy choices in relation to nutrition and physical activity [11], all of which might contribute to elevated CVD risk in this population. Conversely, authors have suggested that in developing countries or affluent countries that high SES is associated with increased CVD risk [12]. This is in part due to greater ability to purchase higher density foods [12] and lower levels of physical activity or increased sedentary behaviours, particularly video game play and television viewing [4,13]. Recent research by Buchan *et al.* [14] identified that the different independent risk factors for CVD were associated with SES in different ways, depending on the risk factor being examined. They noted that high fat diets, low levels of physical activity elevated C-Reactive protein and higher levels of blood cholesterol were the factors most likely to be associated with CVD risk, irrespective of SES, but that elevated CVD risk is seen in both high and low SES adolescents who exhibit poor nutritional behavior and low levels of physical activity [14].

The equivocal nature of the extant data relating to differences in CVD risk between socioeconomic status groups is not unexpected, as this association varies depending on the societal structure in place, the overall economic development of the geographical area being examined, as well as other cultural factors that may impact on a particular SES group in any particular country [7,9,11,15]. It is likely that any relationship between SES and CVD risk is dynamic and will vary across countries. While there is considerable information in relation to the United States [8] and the United Kingdom [15], fewer data are available for other countries, including Portugal [16]. However, the majority of studies have evidenced that in developed countries low SES is associated with greater CVD risk [8,15], unlike low or middle income countries, where the reverse is true [12]. As CVD begins in childhood [17], there is need to understand how factors such as SES impact on these disease processes specific to individual countries of geographical areas so preventive interventions can be targeted. The aim of this study was to examine any SES differences in CVD risk in Portuguese children.

## 2. Methods

### 2.1. Participants

A total of four secondary schools in the Porto district were invited to participate in this study. Details on the study design and sampling strategy are reported elsewhere [18]. Following informed consent, 359 adolescents (202 girls and 157 boys) aged 10 to 17 years (mean age ± SD = 13.9 ± 1.98 years) participated in the study following ethics approval by the faculty and the Portuguese Foundation for Science and Technology ethics committees.

### 2.2. Anthropometric Measures

Height (to the nearest millimeter) and body mass (to the nearest 0.1 kg) were assessed using a stadiometre (Holtain Ltd., Crymmych, Pembrokeshire, UK) and weight scale (Seca 708 portable digital beam scale, Hamburg, Germnay) with participants in bare feet and lightly dressed. Body mass index (BMI) was determined in kg/m^2^ units.

### 2.3. Blood Sampling

Capillary blood samples of participants were taken from the earlobe after at least 12 hours fasting to obtain values of plasmatic total cholesterol (TC), high density lipoprotein cholesterol (HDL), triglycerides (TG), and fasting glucose (GLUC). Low density lipoprotein (LDL) was estimated using the Friedewald equation [19]. The blood samples were assayed using a Cholestech LDX*^®^* Analyser (Cholestech Corporation, Hayward, CA, USA) as this analyser is valid for population-based screening of cardiovascular risks factors [20].

### 2.4. Blood Pressure

Blood pressure (BP) was measured using the Dinamap (BP8800) adult/pediatric and neonatal vital signs monitors (GE Healthcare, Little Chalfont, UK). Measurements were taken in seated posture after at least 5 min rest. Two measurements were taken and the mean of these two measurements was used for statistical analysis following procedures established previously [21].

### 2.5. Measurement of Cardiorespiratory Fitness

Cardiorespiratory fitness (CRF) was predicted using the multistage 20 m shuttle-run test [22] due to its significant correlation with VO_2_max (r = 0.80) in young people [23]. Participants were familiarized with the procedure for each test before recording data and received verbal encouragement from the investigators throughout with VO_2_ max being estimated from the number of shuttles performed.

### 2.6. Cardiovascular Risk Score

Given the lack of a universal definition for CVD risk in children and adolescents [24] we decided to compute a continuous risk score from the following measurements: TC; GLU; HDL; LDL; TG and systolic blood pressure (SBP). For each of these variables, a z-score was computed as the number of SD units from the sample mean after normalization of the variables, *i.e.*, z = ([value – mean]/SD). The HDL-C z-score was multiplied by –1 to indicate higher CVD risk with increasing value. z scores by age were computed for all risk factors. Then, CVD risk was constructed by summing the z scores of all individual risk factors. A lower CVD risk score is indicative of a better overall CVD risk factor profile. The basis for the construction for the CVD risk score was based on prior studies which have used a similar methodology of combining measures independently related to CVD risk and combining these as z-scores, similar to the idea proposed by Andersen *et al.* [25]. However, different authors have used a different combination of measures in constructing their CVD risk scores [3,7,15,18,25]. As there is no current consensus as to which variables best relate to CVD risk the process described above was based on using variables identified by Friedemann *et al.* [6] as being directly related to CVD risk in children.

### 2.7. Socioeconomic Status

Parent’s educational level (PEL) was employed as a proxy for SES, using categories based on the Portuguese Educational System: (1) 9 years education or less—subsecondary level; (2) 10–12 years education—secondary level and (3) College/Master/Doctoral degree—higher education level. These three levels were classified as low, middle and high levels of education, similar to procedures that have previously been applied in the Portuguese context [26,27].

### 2.8. Statistical Analysis

A 2 (gender) by 3 (PEL) ways analysis of covariance (ANCOVA), controlling for BMI and CRF was used to examine any differences in CVD risk score across gender and SES groups whilst controlling for weight status and cardiorespiratory fitness. In the present study BMI was used as a covariate in the analysis as BMI has been associated with CVD risk in children independent of the other variables assessed in this study. The use of ANCOVA in the analysis also enables any differences between CVD risk between PEL groups to be analysed controlling for any impact of weight status, whilst at the same time enabling the association between the dependant variable (CVD risk) and the covariate to be determined [28]. Separate repeated measures ANOVAs were also employed for individual variables within the CVD risk score to examine whether there were any differences in these variables between PEL groups. Where any significant differences were detected Bonferroni post-hoc multiple comparisons were used to assess where the differences lay. Statistical significance was set at 0.05 and the Statistical package for Social Sciences (SPSS, Version 18, SPSS Inc., Chicago, IL, USA) was used for all analysis.

## 3. Results

**Figure 1:**
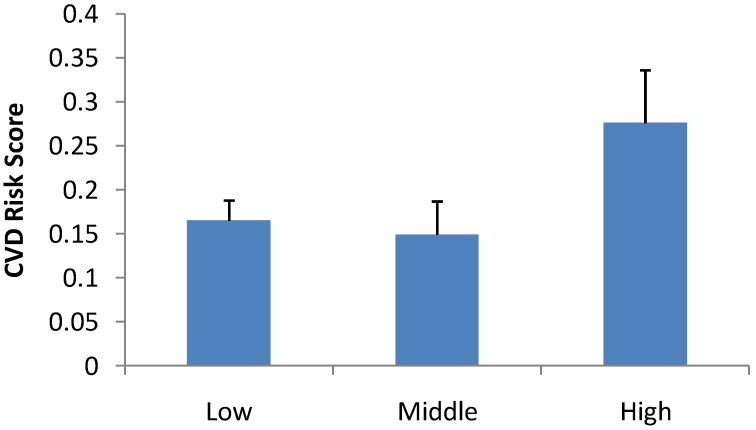
Mean ± S.E. of CVD risk score of Portuguese children with low, middle and high level of parental education.

**Figure 2:**
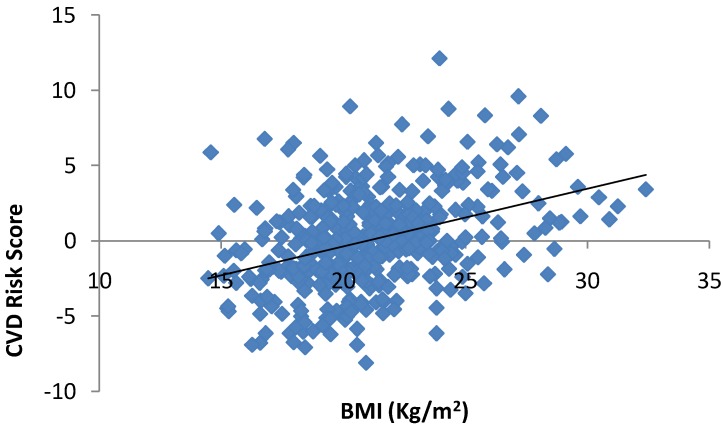
The association between BMI and CVD risk in Portuguese children.

Results from the ANCOVA for CVD Risk indicated that there were no higher order interactions and there was no significant gender main effect (*p* > 0.05). There was a significant main effect for PEL (*p* = 0.019). Bonferroni post hoc multiple comparisons indicated that CVD risk score was significantly lower in low (*p* = 0.024) and middle (*p* = 0.005) PEL compared to high PEL (see Figure 1). There was no significant difference in CVD risk score between low and middle PEL (*p* > 0.05). Moreover, the covariate, BMI was also significant (*p* = 0.0001, β = 0.023) evidencing a significant positive association between BMI and CVD risk with higher BMI associated with greater CVD risk (see Figure 2). CRF was not significant as a covariate (*p* > 0.05). Mean ± S.D. of individual components of the summed CVD risk score acoording to PEL groups is presented in Table 1. Repeated measures ANOVAs for each of the component variables that compried the CVD risk score also indicated significantly higher TC in High PEL compared to middle PEL Level children (*p* = 0.02). LDL was also significantly higher in the high PEL compared to the low (*p* = 0.045) and middle (*p* = 0.015) PEL group. The same trend was also evident for GLUC with values being significantly higher in high PEL children compared to low (*p* = 0.045) and middle PEL (*p* = 0.039) children. Values for CRF were also significantly lower for children from middle PEL compared to those from a low PEL (*p* = 0.027).

**Table 1:** Mean ± S.D. of individual components of the summed CVD risk score acoording to SES groups (* inciates *p* = 0.05 or better).

	SES						
	Low		Middle		High		
	M	S.D.	M	S.D.	M	S.D.	
BMI (kg/m^2^)	21.1	2.79	21.6	3.1	20.8	2.7	
GLUC (mg/dL)	84.8	7.2	84.4	6.2	87.3	8.4	*
TC (mg/dL)	148.3	25.8	144.8	28.3	157.5	27.9	*
HDL (mg/dL)	45.2	11.8	45.3	11.3	45.2	9.8	
LDL (mg/dL)	91.6	24.2	88.4	26.2	100.4	27.7	*
TG (mg/dL)	57.6	20.1	52.2	15.4	57.1	19.5	
SBP (mmHg)	122.6	13.8	125.8	13.1	123.6	12.3	
CRF (mL/kg/min^−1^)	48.1	3.8	46.7	3.6	47.4	3.7	*

## 4. Discussion

This study presents data on PEL differences in CVD risk in Portuguese children and adolescents. The results of this study suggest that PEL impacts CVD risk in this population. Making the assumption that PEL is a reflection of SES, the current data support assertions made previously using a variety of other means to determine SES [9], but refute conclusions made by other authors [15]. SES is a dynamic variable that changes over time and across countries and environments and it is therefore likely that any effect of SES on CVD risk (or other health indices) needs to be considered in the context of the location where such data were gathered. In this instance, children with parents who had a higher education level exhibited higher CVD risk. This is contrary to studies from the US [8], which reported lower SES to be related to greater CVD risk. However, the current results are congruent with studies from Brazil [29,30]. Such SES differences are largely attributable to lifestyle factors such as consumption of more energy dense diets and higher BMI, leading to increased CVD abnormality [8]. Moreover, food availability (in both quantity and quality) at extremes of SES is also a consideration in terms of the data presented here with higher risk being seen in high SES and low SES (although not statistically significant) children in this study.

The covariate “BMI” was also significantly associated with CVD risk in the present study. This finding is not surprising and the association between measures of overweight/obesity and CVD risk have been reported previously [7]. Furthermore, BMI has been established as an independent risk factor for CVD in children and prior research suggested it should be analysed alongside, but separate to other measures of CVD risk [6]. Thuis the current study not only evidences the association between BMI and CVD risk in Portuguese children and adolescents, but evidences that there are differences in CVD risk scores between PEL groups when weight status is controlled for within statistical analysis.

However, the data presented here are cross sectional and this limits our ability to determine causality. There is also a need to understand how SES might influence other variables, including physical activity, in relation to CVD risk in young people. The present study is therefore limited because no information other than SES, weight status and physical fitness was used. This should be considered a limitation and future studies should also look to assess or control for confounding variables in the SES-CVD relationship, such as diet and physical activity. This study was exploratory in nature and additional research is needed which examines a wider range or variables including dietary and physical activity practices of Portuguese children from different SES groups is needed to more fully understand how SES influences CVD risk. Moreover, a CVD risk score was calculated in the present study using summed z-scores from a range of variables independently related to cardiovascular disease in children. This approach was taken following procedures previously used to indentify CVD risk in children [3,7,18]. However, the most appropriate range of measures best placed to capture CVD risk in children as a continuous variable is not currently known [6,14]. Furthermore, it may have been useful to have employed recognized cut-off points for related disease states (e.g., metabolic syndrome using NCEP cut-points) in this analysis. However, this approach would have not enabled use of a continuous risk score. We therefore acknowledge that the CVD risk presented here is a reflection of the measures combined to create the score. Although the use of a continuous CVD risk score has been recommended [6] future research would be welcome which determines which combination of variables best reflect overall CVD risk in children and/or uses other established cut-offs to illustrate the CVD risk-SES relationship. Finally, the use of parental education level, although used by prior studies [26] as a proxy for SES, is not without criticism and future researchers would benefit from incorporating multiple indices of SES into one scale in order to provide a more sensitive measure of SES than that used in the current study. Although prior studies on Portuguese children and adolescents have used this method [13,17] and the acknowledgement that there is no gold standard for the assessment of SES in young people [31], the use of parental education as a measure of SES infers child SES based on their parent’s estimated SES. This method also ignores a number of other key variables related to SES, and others [32] have suggested that more precise measures of child and adolescent SES may be better based on indices of multiple deprivation rather than relying solely on one variable such as is the case in the present study. Furthermore, the method of establishing SES in the current study might also explain some of the findings with children of highly educated parents presenting higher CVD risk, presumably due to a high education leading to greater income and thus more ability to purchase higher density food, greater access to motorized personal transport and electronic equipment associated with sedentary behavior. We do however acknowledge that this suggestion is speculative and further research would be needed to substantiate these claims.

## 5. Conclusions

This study examined the impact of PEL on CVD risk in Portuguese children. In this study, significantly greater CVD risk was found for children of high PEL after controlling for weight status and cardiorespiratory fitness. Thus, health related interventions are needed which target this group specifically. Further research is needed to elucidate the lifestyle variables that result in this difference risk in Portuguese children.
